# Criteria and potential predictors of severity in patients with COVID-19

**DOI:** 10.1186/s43168-022-00116-y

**Published:** 2022-02-19

**Authors:** Niveen E. Zayed, Ahmad Abbas, Samah Mohamad Lutfy

**Affiliations:** Chest Department, Faculty of Medicine, Zagazig University, Zagazig, 44519 Egypt

**Keywords:** COVID-19, Ferritin, ICU, Mean platelet volume

## Abstract

**Background:**

The challenge in treating severe COVID-19 in the absence of targeted medication is enforcing physicians to search carefully for clinical predictors of severity.

**Aim:**

To define the profile of patients at risk of severe COVID-19 and to assess for certain predictors.

**Methods:**

Confirmed COVID-19 cases were classified into the following: group A: mild/moderate cases and group B: severe/critical cases according to the selected criteria. History, radiological assessment, complete blood count, lactate dehydrogenase (LDH), myocardial enzymes, serum ferritin, and D dimer were assessed. Patients were followed for the need of ICU and mechanical ventilation. Duration till conversion, length of stay, and mortality were recorded.

**Results:**

A total of 202 patients were analyzed. Group B had higher age (53.2 ± 12.6 vs 40.3 ± 10.3, *P* < 0.001), more prevalence of DM (60.61% vs 16.57% *P* < 0.001), hypertension (51.52% vs 20.12%, *P* < 0.001), ischemic heart (27.27% vs 3.55%, *P* < 0.001), bronchial asthma (36.36% vs 3.55%, *P* < 0.001), COPD (9.09% vs 1.18%, *P* = 0.03), higher mean platelet volume (MPV) (12.76 ± 7.13 vs 10.51 ± 7.78 (fL), *P* < 0.001), higher serum ferritin (954 ± 138 vs 447 ± 166 ng/ml, *P* < 0.001), higher LDH (604 ± 220 vs 384 ± 183 U/L, *P*-value < 0.001), higher creatine phosphokinase (24.27 ± 5.82 vs 16.4 ± 4.87 IU/L, *P* < 0.001), and higher mortality (30.3% vs 0.6%, *P* < 0.001). Multivariate regression of predictors of severity identified three predictors; age, MPV, serum ferritin, and IHD.

**Conclusions:**

The current study places of interest the characteristic host-related features of severe COVID-19 and draws attention to potential predictors.

## Introduction

An outbreak of pneumonia of unidentified etiology was discovered in Wuhan city, China, in December 2019; the scientist succeeded in early January 2020 to isolate a novel virus which seems to belong to the genus *Betacoronavirus*, Coronaviridae family. They gave them a name, SARS-CoV-2 [1–4]. Later on, WHO gave the disease its unique name, COVID-19, and announced it as a pandemic by 11 February 2020 [5, 6].

The most common presentation of COVID-19 pneumonia is fever, dry cough, and dyspnea, which may be associated with fatigue and myalgia. Most of the cases had a mild to moderate course of disease and recovered after good medical intervention. A small number of patients may develop severe pneumonia, 15–32%, with acute respiratory distress syndrome (ARDS) and multiple organ failure, or even death [5, 7–10].

The fatality rate ranged from 1 to 15% [11]. The challenge in treating severe and critically ill cases with COVID-19 especially with the absence of specific and targeted medication enforcing the physician to search carefully for clinical characteristics of severe COVID-19 pneumonia and subsequent predictors in order to start the optimum standard of care as early as possible. Growing reports highlighted old age, cardiovascular comorbidity, and diabetes mellitus as predictors of severe course of disease [12]. Others identified several biomarkers to help in predicting severe and fatal coronavirus disease 2019 (COVID-19) such as serum ferritin, D-dimer, and cardiac enzymes [13]. The aim of this work is to define the profile of patients at risk of severe COVID-19 and to assess for certain predictors of severity.

## Patients and methods

This is an observational cross-sectional study including 202 patients diagnosed to have COVID-19. The study was conducted at Zagazig University isolation hospitals from the period of March 2020 to June 2021. The study was approved by Zagazig University Ethics Committee (number 9229). Written informed consent was obtained from all participants.

### Inclusion criteria

The study includes laboratory-confirmed COVID-19 patients (confirmed by real-time polymerase chain reaction) admitted l during the period of the study.

The patients were classified into two groups: group A included mild/moderate cases and group B included severe/critical cases according to the following criteria.

Severity of COVID-19 was graded as follows: (1) mild: mild clinical symptoms, no pneumonia on lung CT; (2) moderate: fever, cough, and lung CT with pneumonia; (3) severe: respiratory distress (respiratory rate > 30/min, oxygen saturation (O2Sat) ≤ 93% at rest and/or ratio of arterial oxygen partial pressure to fractional inspired oxygen ≤ 300 mmHg (PaO_2_/FIO_2_); and (4) critical: aforementioned criteria of respiratory failure receiving mechanical ventilation, shock, and/or organ failure other than lung and/or intensive care unit (ICU) hospitalization [14, 15].

All participants were subjected to full history taking including smoking history and comorbidity profile. Clinical symptoms including fever, cough, dyspnea, myalgia, hemoptysis, sore throat, diarrhea, loss of smell, and anorexia were recorded. Radiological assessment by initial chest x-ray then CT chest as possible and appropriate. Routine laboratory investigations: complete blood count (CBC), coagulation profile, serum biochemical tests (including renal and liver function, and electrolytes), laboratory investigation to assess the severity of COVID-19: lactate dehydrogenase (LDH), myocardial enzymes (CPK-MB), serum ferritin, D dimer, and arterial blood gas analysis. Patients were followed up for the need for ICU and mechanical ventilation (MV), duration till conversion, total length of stay, and final outcome whether survived or died were recorded.

### Statistical methods

The statistical analysis was done using Minitab 17.1.0.0 for Windows (Minitab Inc., 2013, Pennsylvania, USA). The data normality was examined using the Shapiro-Wilk test, the continuous data represented as mean and standard deviation (SD), and categorical data as number and percentage (%). Comparison between two means was done using independent t test, while chi-square test compared the frequency of two groups or more. Logistic regression analysis test with backward elimination methods was used to predict the factors associated with severity. The accuracy of MPV and ferritin were assessed using receiver operating curve analysis; assuming that the area under the ROC curve of 0.9 for this study was significant with margin of type I error 0.05 and type II error 0.1, the sample size calculated with a minimum total number of 70 and the minimum number of cases with severe COVID-19 was 35 using SigmaPlot software 12.5.0.38 for Windows (SigmaPlot, Systat Software Inc. UK, 2011). All tests were two sided, *P* considered significant if < 0.05.

## Results

**Fig. 1:**
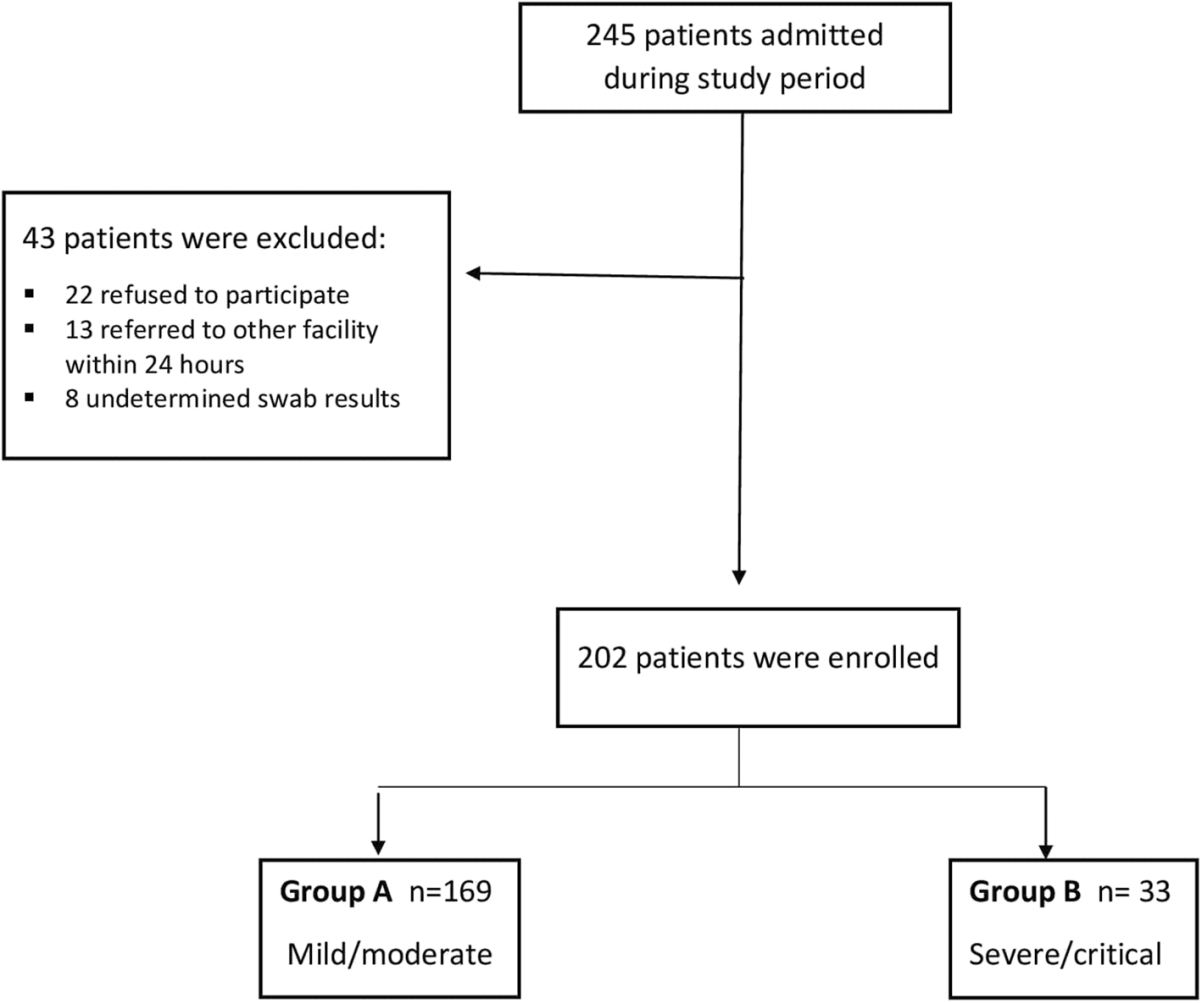
Flow chart of the studied population

This study analyzed 202 patients proved to have COVID-19 by real time PCR, they were classified into two groups, group A with mild/moderate COVID-19 and group B with severe/critical COVID-19 (Fig. 1) according to the aforementioned criteria. Table 1 demonstrates the demographic data of both groups where group B had higher age (53.2 ± 12.6 vs 40.3 ± 10.3, *P* < 0.001), more prevalence of comorbidity profile, DM (60.61% vs 16.57% *P* < 0.001), hypertension (51.52% vs 20.12%, *P* < 0.001), ischemic heart disease (IHD) (27.27% vs 3.55%, *P* < 0.001), bronchial asthma (36.36% vs 3.55%, *P* < 0.001), COPD (9.09% vs 1.18%, *P* = 0.03), and hyperlipidemia (12.12% vs 2.37%, *P* = 0.01).

**Table 1:** Demographic data of the studied COVID-19 patients

Variables	Mild & moderate (*n* = 169)	Severe & critical (*n* = 33)	*P*-value
Age	40.3 ± 10.3	53.2 ± 12.6	< 0.001*
Sex (male)	133 (78.7%)	29 (87.9%)	0.23^‡^
Smoking	48 (28.4%)	12 (36.4%)	0.36^‡^
Comorbidities			
DM	28 (16.6%)	20 (60.6%)	< 0.001^‡^
Hypertension	34 (20.1)	17 (51.5%)	< 0.001^‡^
IHD	6 (3.6%)	9 (27.3%)	< 0.001^‡^
Bronchial asthma	6 (3.6%)	12 (36.4%)	< 0.001^‡^
COPD	2 (1.2%)	3 (9.1%)	0.03^‡^
Hyperlipidemia	4 (2.4%)	4 (12.1%)	0.01^‡^
Hypothyroidism	2 (1.2%)	0 (0%)	1^‡^

Continuous data represented as mean and SD, and categorical data represented as number and percentage (%).*Independent samples Student’s *t*-test; ^‡^chi-square test; *P* < 0.05 is significant; *DM*, diabetes mellitus; *IHD*, ischemic heart disease; *COPD*, chronic obstructive pulmonary disease

The clinical characteristics of the studied COVID-19 patients are shown in Table 2, which revealed that group B had longer duration of symptoms before admission (5.55 ± 2.15 days vs 3.89 ± 1.1 days, *P* < 0.001). Regarding the presenting symptoms, group B had more prevalence of dyspnea (81.82% vs 37.28%, *P* < 0.001), cough (100% vs 83.43%, *P* = 0.012), myalgia (66.67% vs 46.15%, *P* = 0.03), hemoptysis (24.24% vs 0%, *P* < 0.001), diarrhea (72.73% vs 43.2%, *P* = 0.002), loss of smell (48.5% vs 29.59%, *P* = 0.034), anorexia (39.4% vs 21.3%, *P* = 0.27).

**Table 2:** Clinical characteristics of the studied COVID-19 patients

Variables	Mild & moderate (*n* = 169)	Severe & critical (*n* = 33)	*P*-value
Duration of symptoms before admission	3.9 ± 1.1	5.6 ± 2.2	< 0.001*
Fever	151 (89.4%)	30 (90.9%)	0.79^‡^
Cough	141 (83.4%)	33 (100%)	0.012^‡^
Expectoration	16 (9.5%)	0 (0%)	0.07^‡^
Dyspnea	63 (37.3%)	27 (81.8%)	< 0.001^‡^
Myalgia	78 (46.2%)	22 (66.7%)	0.03^‡^
Sore throat	59 (34.9%)	9 (27.3%)	0.39^‡^
Hemoptysis	0 (0%)	8 (24.2%)	< 0.001^‡^
Diarrhea	37 (43.2%)	24 (72.7%)	0.002^‡^
Loss of smell	50 (29.6%)	16 (48.5%)	0.034^‡^
Anorexia	36 (21.3%)	13 (39.4%)	0.027^‡^

Continuous data represented as mean and SD, and categorical data represented as number and percentage (%).*Independent samples Student’s *t*-test; ^‡^chi-square test; *P* < 0.05 is significant

Regarding the radiological presentation, abnormalities were bilateral in all cases of group B vs 71.6% in group A with *P*-value < 0.001, more reticular infiltrates 36.4% in group B vs 10.6% in group A with *P*-value 0.02 and more ground-glass opacities (GGO) 72.73% in group B vs 19.53% in group A with *P*-value < 0.001 as shown in Table 3.

**Table 3:** Chest x-ray and CT abnormalities of studied COVID-19 patients

Variables	Mild & moderate (*n* = 169)	Severe & critical (*n* = 33)	*P*-value
Radiological infiltrates (yes)	121 (71.6%)	33 (100%)	< 0.001^‡^
Bilateral	77 (45.6%)	33 (100%)	< 0.001^‡^
Nodular	10 (5.9%)	2 (6.1%)	0.76^‡^
Reticular	18 (10.6%)	12 (36.4%)	0.02^‡^
GGO	32 (19.5%)	24 (72.7%)	< 0.001^‡^

Categorical data represented as number and percentage (%). ^‡^chi-square test; *P* < 0.05 is significant; *GGO*, ground-glass opacity

Laboratory assessment of patients on admission is shown in Table 4, which revealed the followings in group B compared to group A; more lymphopenia (16.18% ± 7.13% for group B vs 42.1% ± 7.78% for group A *P* < 0.001), higher MPV ( 12.76 ± 7.13 (fL) vs 10.51 ± 7.78 (fL), *P* < 0.001), higher ESR (60.4 ± 17.7 (mm/h) vs 39 ± 19.6 (mm/h), *P* < 0.001), higher CRP (119.5 ± 45.6 mg/L vs 59.6 ± 39.5 mg/L, *P* < 0.001), higher serum ferritin (954 ± 138 ng/ml vs 447 ± 166 ng/ml, *P* < 0.001), more positive D-dimer (75.8% vs 20.7%, < 0.001), higher LDH (604 ± 220 U/L vs 384 ± 183 U/L, *P*-value < 0.001), higher AST (69 ± 37 U/L vs 51.7 ± 25.4 U/L, *P* = 0.01), higher ALT (59.5 ± 17.7 U/L vs 48.2 ± 26.5 U/L, *P* = 0.01), higher creatinine (1.25 ± 0.65 mg/dL vs 0.83 ± 0.2mg/dL, *P* = 0.01), and higher CPK-MB (24.27 ± 5.82 IU/L vs 16.4 ± 4.87 IU/L, *P* < 0.001).

**Table 4:** Laboratory assessment of the studied COVID-19 patients on admission

Variables	Mild/moderate (*n* = 169)	Severe/critical (*n* = 33)	*P*-value
**Laboratory investigation**			
TLC	5.97 ± 1.92	6.7 ± 3.4	0.19*
Platelet	201.3 ± 61.9	187.5 ± 70.2	0.23*
HgB	15.3 ± 1.6	14.2 ± 2.6	0.02*
%Lymph	42.1 ± 7.8	16.2 ± 7.1	< 0.001*
MPV	10.5 ± 1.8	12.8 ± 7.1	< 0.001*
ESR	39 ± 19.6	60.4 ± 17.7	< 0.001*
CRP	59.6 ± 39.5	119.5 ± 45.6	< 0.001*
Ferritin	447 ± 166	954 ± 138	< 0.001*
Positive D-dimer	35 (20.7%)	25 (75.8%)	< 0.001^‡^
LDH	384 ± 183	604 ± 220	< 0.001*
AST	51.7 ± 25.4	69 ± 37	0.01*
ALT	48.2 ± 26.5	59. 5 ± 17.7	0.01*
Creatinine	0.8 ± 0.2	1.3 ± 0.7	0.01*
CPK-MB	16.4 ± 4.9	24.3 ± 5.8	< 0.001*
**ABG**			
PH	7.4 ± 0.3	7.4 ± 0.6	0.06
PCO_2_	37.4 ± 3.6	32 ± 3.9	< 0.001*
PO_2_	70.8 ± 9.3	49.6 ± 2.6	< 0.001*
HCO_3_	20.9 ± 1.97	19.6 ± 2.8	0.03*
Na	131.7 ± 4.5	129.7 ± 4.7	0.03*
K	3.5 ± 0.4	3.2 ± 0.6	0.02*

Continuous data represented as mean and SD, and categorical data represented as number and percentage (%).*Independent samples Student’s *t*-test; ^‡^chi-square test; *P* < 0.05 is significant; *TLC*, total leucocyte count (cell/μL)^3^; *PLT*, platelet count (cell/μL)^3^; *HgB*, hemoglobin (gm/dL); %Lymph (cell/μL)^3^; *MPV*, mean platelet volume (fL); *ESR*, erythrocyte sedimentation rate (mm/h); *CRP*, C-reactive protein (mg/L); ferritin (ng/ml); D-dimer, (mg/L); *LDH*, lactate dehydrogenase (U/L); *AST*, aspartate transaminase (U/L); *ALT*, alanine aminotransferase (U/L); creatinine (mg/dL); *CPK-MB*, creatine kinase myocardial band (IU/L); *PCO*_*2*_, partial pressure of arterial carbon dioxide (mm HG); *PO*_*2*_, partial pressure of arterial oxygen (mm HG); *Na*, sodium (mEq/L); *K*, potassium (mmol/L)

Group B also had more hypoxemia than group A (49.58 ± 2.59 mmHg vs 70.77±9.31 mmHg respectively, *P* < 0.001) in ambient air, lower PCO_2_ (32.01 ± 3.94 mmHg vs 37.41 ± 3.56 mmHg respectively, *P* < 0.001), lower Na (129.7 ± 4.7mEq/L vs 131.72 ± 4.5 mEq/L, respectively, *P* = 0.03) and lower K (3.19 ± 0.6 mmol/L vs 3.46 ± 0.43 mmol/L respectively, *P* = 0.02).

Regarding the outcome of both groups; group B had longer duration till conversion (9.44 ± 2.6 vs 7.88 ± 2.5 days, *P* = 0.03), longer length of hospital stays (10.7 ± 3.13 vs 7.6 ± 1.65 days, *P* = 0.001), more ICU admission (100% vs 30.18%, *P* < 0.001), more need of MV (87.9% vs 2.37%, *P* < 0.001) and higher mortality rate (33.3% vs 0.6%, *P* < 0.001) as shown in Table 5.

**Table 5:** Outcome of the studied COVID-19 patients

Variables	Mild/moderate (*n* = 169)	Severe/critical (*n* = 33)	*P*-value
Duration till conversion	7.88 ± 2.5	9.4 ± 2.6	0.03*
LOS	7.6 ± 1.7	10.7 ± 3.1	0.001*
Need ICU	51 (30.2%)	33 (100%)	< 0.001^‡^
Need MV	4 (2.4%)	29 (87.9%)	< 0.001^‡^
Death	1 (0.6%)	10 (33.3%)	< 0.001^‡^

Continuous data represented as mean and SD, and categorical data represented as number and percentage (%).*Independent samples Student’s *t*-test; ^‡^chi-square test; *P* < 0.05 is significant; *LOS*, length of stay

Multivariate regression of potential predictors of COVID-19 severity is shown in Table 6 and revealed the following predictors: age (OR = 1.542, CI = 1.21–1.93), MPV (OR = 6.67, CI = 1.32–3.68), serum ferritin (OR = 2.04, CI *=* 1.19–3.47), and IHD with (OR = 3.1, CI = 1.25–4.94).

**Table 6:** Predictors of severity of COVID-19 patients using regression analysis

Variables	OR	95% CI	*P*-value
Age	1.5	(1.21–1.93)	0.044
MPV	6.7	(1.32–3.68)	0.004
Ferritin	2.04	(1.2–3.5)	0.016
IHD	3.1	(1.3–4.9)	0.003

Pearson, *X*^2^ = 9.32, *P* = 0.316

**Fig. 2:**
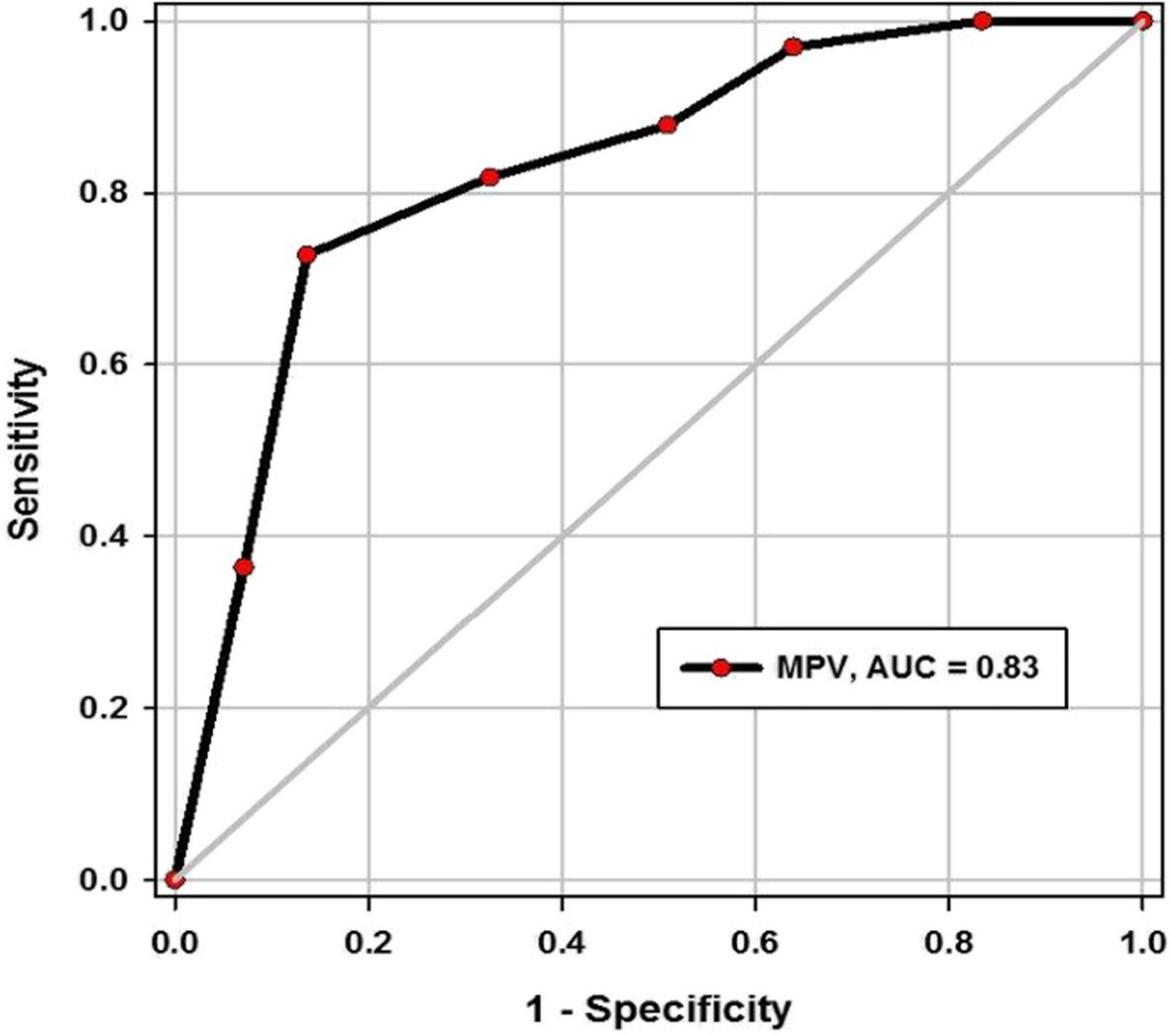
Receiver operating characteristic (ROC) curve for MPV in predicting severity of COVID-19

**Fig. 3:**
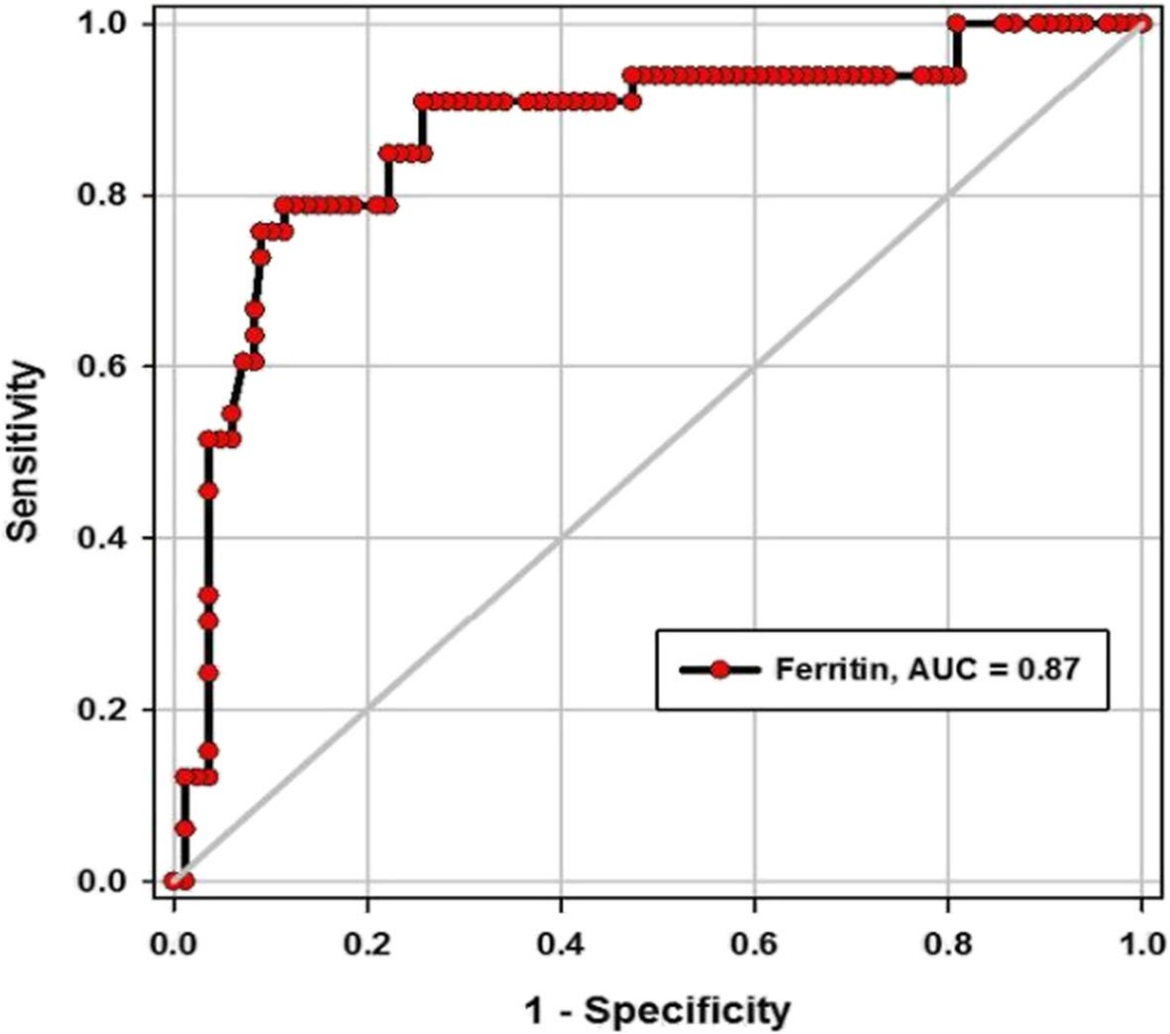
Receiver operating characteristic (ROC) curve for serum ferritin in predicting severity of COVID-19

Table 7 and Figs. 2 and 3 report the overall results of the ROC analysis regarding MPV and serum ferritin as predictors of COVID-19 severity. A cutoff of ferritin > 548.5 ng/ml yielded 91% sensitivity, 71% specificity, 43% positive predictive value (PPV), 97% negative predictive value (NPV) (AUC, 0.87). A cutoff of MPV > 9.5 (fL) yielded 96% sensitivity, 63% specificity, 25% positive predictive value (PPV), 98% negative predictive value (NPV) (AUC, 0.83).

**Table 7:** Validity of ferritin and MPV in predicting COVID-19 severity

Variable cutoff	Sensitivity (95%CI)	Specificity (95%CI)	PPV (95%CI)	NPV (95%CI)	AUC (95%CI)	*P*-value
Ferritin > 548.5 ng/ml	91% (75.6–98.1)	74% (66.9–80)	43% (19.7–51.5)	97% (84.8–99.1)	0.87 (0.79–0.94)	< 0.001
MPV > 9.5 fL	96% (84.2–99)	63% (29.2–44.3)	25% (16.3–35.8)	98% (85.3–99.5)	0.83 (0.75–0.9)	< 0.001

## Discussion

The aspects of COVID-19 disease are widely ranging from asymptomatic condition up to multi-organ dysfunction. The clinical presentation cannot be discriminated from other respiratory diseases; hence, they share the common features such as fever, headache, fatigue, sore throat, cough, and dyspnea [7].

As mentioned in published reports, about 20 to 30% of patients progress to have pneumonia that need hospitalization and could be deteriorating to acute respiratory distress (ARDS) and death [16].

The host-related factors such as older age and presence of comorbidities play a significant role in determining the course of disease [17], which support the current data, as the elderly patients with DM, hypertension (HTN), ischemic heart disease (IHD), and chronic respiratory disease significantly suffered from severe disease. In the same line, Du et al. [18] found age above 65 years with cardiovascular and cerebrovascular comorbidities associated with severe COVID-19 diseases. Moreover, He et al. [19] added that patients with chronic kidney disease had severe a COVID-19 course.

A recent meta-analysis summarizes the linked host-related risk factors and concluded that severe COVID-19 patients are more likely to be older with associated different cardiovascular and respiratory comorbidities and explained that by weak immune function [20]. However, in other studies, the risk factors were different and included male sex, smoking habits, and obesity [21, 22].

Considering the radiological workup, the present work highlights in brief the role of CT chest in characterizing severe COVID-19 patients; hence, they significantly presented with a bilateral lesion, mostly GGO and reticulation. Many studies support our finding; thus, CT plays a significant role in diagnosing the pulmonary changes associated with SARS-CoV-2 infection with higher sensitivity action [7].

Like other respiratory viruses, SARS-CoV-2 infection implies different hematological changes, which help in the evaluation and monitoring of disease severity. The commonest changes in the hematopoietic system are lymphocytopenia, neutrophilia, thrombocytosis, or thrombocytopenia; therefore, during the course of the disease, the total leukocyte count may show normal value, decrease, or increase [23]. The present data recorded that the relative lymphocyte count was significantly decreased in the severe group rather than the mild and moderate ones, while total leucocytic count (TLC) and platelet (PLT) count showed insignificant differences that came in agreement with other reports; thus, the inflammatory cells were consumed during the immune response [24, 25]. On the other hand, a contrary study recorded elevated TLC and PLT count in severe COVID-19 [19].

Taking into consideration other laboratory findings, we reported elevation in ESR, CRP, LDH, AST, ALT, CPK-MB, D-dimer, and creatinine levels in severe COVID-19 patients, which came in accordance with previous studies. In consequence, the hypercoagulable state is implicated in the formation of tiny thrombi in different organs, which later on promote a bad prognosis. Accordingly, the tests of coagulation profile help in assessing the critical patients as well as guiding the treatment intervention [26–28].

Considering the role of ferritin and its role during immune response toward infection, the idea focused on the mandatory function of iron in viral replication inside the cells; moreover, iron increases the sensitivity of T lymphocytes toward the chemical mediators, which finally helps in the regulation of ferritin expression. Ferritin plays an important immune modulator action by its heavy and light chains (HFC and LFC), through binding to iron molecules; consequently, the biological available form of iron protects the cellular proteins, lipids, and DNA from the toxicity of heavy metals. As a response to the inflammatory process toward viral infection including SARS-CoV-2, a significant elevation of serum ferritin level was noticed, as the pro-inflammatory cytokines particularly IL-6 stimulate the production of hepcidin, which upregulates the level of ferritin [29–32].

Our data showed significant elevation of the ferritin level in severe COVID-19 patients, and its sensitivity in predicting disease severity reached 91% at cutoff level > 548.5 ng/mL. In addition, it could be used as independent predictors of severe COVID-19, and so, the likelihood of severity increased twofold more with every unit increase in the ferritin level; OR = 2.04, *P* = 0.016. Using ferritin as a biomarker for severe SARS-CoV-2 infection was discussed by a recent publication, especially in Asian areas with a recommendation of empowering the study by more observations, as the finding was not conclusive; hence, the level of ferritin was elevated in different types of infections and in all COVID-19 patients [33].

On the other hand, Velavan et al. and Mehta et al. considered ferritin a significant predictor of severe COVID-19 disease and mortality due to secondary hemophagocytic lymphohistiocytosis (sHLH) and cytokine storm syndrome. Another important index involved in evaluating inflammatory condition was MPV; it correlates with platelet activation and reflects prothrombotic conditions [34–36].

The role of platelets in inflammation and immune regulation is crucial; hence, the activated platelets in viral infection induced lung injury through different mechanisms; first, direct damage of respiratory tract by released inflammatory mediators; second, surface exposure of E and P selectin that attract other inflammatory cells causing inflammatory and immune responses. The MPV is an important platelet index that represents platelet activation and indirectly correlates with platelet count. The decrease of platelet count is associated with poor outcome even mortality as well as the increase of MPV [37–40].

In our study, the platelets count was lower in the severe group than in the mild and moderate ones but with insignificant differences; in spite of that, the MPV was significantly higher in the severe group than the mild/moderate one. Moreover, the performance of MPV in predicting severe COVID-19 disease was very good; hence, the AUC was 83%. In addition, the sensitivity of MPV at cutoff point > 9.5 fL was higher (96%). Furthermore, MPV could be used as independent predictors for severe COVID-19, thus the likelihood of severity increased 7-fold for every unit increase of MPV; OR = 6.67, *P* < 0.001.

The study conducted by He et al. [19] showed that platelet count was significantly lower in severe disease, and was considered a predictor for mortality, in disagreement with the present results; hence, the authors neglect the role of the MPV index. However, a limited study discussed the predictive role of MPV in severe COVID-19 disease; a Turkish study examined the correlation between the MPV value in two age groups (above and below 65) [41]. The results suggested irrelevant correlation, and the author recommended a larger study for significant findings. Another retrospective study by Zhong et al. [42] inspected the role of mean platelet volume over platelet count in predicting severe COVID-19 pneumonia and concluded that the capability of that ratio was higher and could be considered independent risk factors.

The strength of the current study is the idea about MPV and ferritin and their predictive ability of severe COVID-19 disease. In spite of the study being a preliminary prospective observation from one medical center, it gave a brief highlight about different independent predictors of the severity of that novel virus.

The limitation was subjected around the lack of other more specific laboratory markers reflecting the inflammatory state and cytokine storm such as IL-1, 6, and 8, TNFα, and CD4 and CD8 counts, but the introduction of common markers that can be easily available was our target. Consequently, further studies are recommended to emphasize the underlying disease pathogenesis.

In conclusion, the study highlights the related aspects of severe COVID-19 disease and draws attention to predictors of severity besides the easy use of available biomarkers such as ferritin and MPV to facilitate the initial detection of a bad prognosis.

## Data Availability

The database used and analyzed during the current study are available from the corresponding author on reasonable request
